# Epidermal Growth Factor Receptor Variant III Mutation, an Emerging Molecular Marker in Glioblastoma Multiforme Patients: A Single Institution Study on the Indian Population

**DOI:** 10.7759/cureus.26412

**Published:** 2022-06-29

**Authors:** Garima Garima, Sharad Thanvi, Anurag Singh, Vijay Verma

**Affiliations:** 1 Pathology, Government Medical College, Pali, Pali, IND; 2 Neurosurgery, Dr. Sampurnanand Medical College, Jodhpur, IND; 3 Pediatrics, Dr. Sampurnanand Medical College, Jodhpur, IND; 4 General Surgery, Dr. Sampurnanand Medical College, Jodhpur, IND

**Keywords:** world health organization classification 2007, genetic pathways, rt-pcr, targeted therapy, egfr mutation variant iii, molecular markers

## Abstract

Background

Glioblastoma is the most frequent and the most aggressive primary malignant brain tumor in adults. Standard treatment includes surgical removal of the tumor followed by concomitant chemotherapy and radiotherapy. Temozolomide, an oral alkylating agent, is currently the most commonly used chemotherapy. However, the median survival of glioblastoma multiforme (GBM) patients remains very low. Epidermal growth factor receptor variant III (EGFRvIII) is a novel marker for GBM patients of Indian origin as very few studies have been done on this molecular marker in our country. This is the first study utilizing this molecular marker among GBM patients in Rajasthan, India. This was a single institutional study that aimed to estimate the proportion of EGFRvIII mutation in GBM patients of Indian origin.

Methodology

This was a non-randomized, ambispective, single institutional observational study done on 35 brain tissue biopsies of histopathologically diagnosed and confirmed cases of GBM based on the World Health Organization 2007 Classification received in the pathology department of Dr. Sampurnanand Medical College, Jodhpur from 2015 to 2020 after applying inclusion and exclusion criteria. Molecular study of the EGFRvIII marker was conducted in all cases of GBM in the same institution on the RNA extracted from selected biopsy samples. Statistical analysis was performed using the SPSS version 22.0 software package (IBM Corp., Armonk, NY USA). The correlation between age and gender with EGFR-positive cases was analyzed, and EGFR positivity compared with previous studies.

Results

The occurrence of the EGFRvIII mutation was found to be 17.4% (6/35 cases). The mean age of presentation of a tumor with this mutation was estimated to be 54.3 years. Males were more commonly found to be affected (66.6%, 4/6 cases).

Conclusions

Thus, the identification of this mutation would segregate patients who may benefit from newer therapeutic approaches. In the future, personalized treatment may be advised for GBM patients depending on the presence of the EGFRvIII mutation.

## Introduction

The epidermal growth factor receptor (EGFR), also known as HER1 or ERBB1, is an important regulator of cellular growth in tissues of epithelial origin. The EGFR gene is the cellular homolog of the v-erbB oncogene originally identified in avian erythroblastosis viruses [1]. In glioblastoma, a particular group of EGFR deletions and point mutations are frequently found. These include EGFRvI (N-terminal deletion), vII (deletions of exons 14-15), vIII (deletions of exons 2-7), vIV (deletions of exons 25-27), and vV (deletions of exons 25-28), among which vII and vIII are oncogenic [1].

Among these mutations, EGFRvIII occurs most frequently with a global incidence of 10-50% [2]. EGFRvIII is 145 KDa in molecular weight [3]. Compared with the EGFR, EGFRvIII has an in-frame deletion of exons 2-7, resulting in a shorter extracellular domain. Compared to the wild-type EGFR, EGFRvIII lacks amino acids 6-273, and deletion of those 267 amino acids creates a junction site with a new glycine residue between amino acids 5 and 274 [4-6]. It mimics ligand binding effects and initiates conformational changes in receptors, leading to the activation of signaling pathways.

Being a constitutively active mutant of EGFR, EGFRvIII transduces signals via traditional EGFR pathways, namely, RAS/mitogen-activated protein kinase (MAPK), phosphoinositide 3-kinase (PI3K)/Akt, and Janus kinase (JAK)/signal transducer and activator of transcription (STAT) pathways. Although EGFRvIII activates several downstream pathways, evidence shows that it preferentially activates the PI3K/Akt signal transduction pathway [6-9]. Recently, EGFRvIII has been found to activate the mammalian target of rapamycin complex 2 (mTORC2) (CREB-regulated transcription co-activator 2) via the PI3K/Akt pathway, which, in turn, leads to stimulation of the nuclear factor-kappa B (NF-κB) pathway, thus leading to resistance to chemotherapy [10]. Another consequence of EGFRvIII activation of PI3K/Akt is increased proliferation and cell cycle progression, which is mediated by a decrease in the level of p27KIP1, a cyclin-dependent kinase (CDK) inhibitor that binds and inactivates cyclin-dependent kinase 2 (CDK2)-cyclin E complexes, thus inhibiting the transition of cells from the G1 to the S phase [11]. The significance of PI3K/Akt activation by EGFRvIII has been confirmed by Klingler-Hoffmann et al., who showed in their study that treatment of U87MG.D2-7 cells with the PI3K inhibitor wortmannin, or by reconstitution of the physiological levels of phosphatase and tensin homolog (PTEN) (a negative regulator of PI3K), resulted in the discontinuation of the EGFRvIII-conferred growth advantage [12]. Selective activation of the PI3K/Akt pathway by EGFRvIII is thought to mediate the resistance to radiation that is observed in EGFRvIII-positive tumors [13-17].

Glioblastoma multiforme (GBM) is a highly proliferative, vascular, and locally invasive tumor [18-20]. Evidence suggests that EGFRvIII-transfected GBM cell lines have higher rates of proliferation, angiogenesis, increased ability to form tumor xenografts, reduced apoptosis, and higher invasiveness when compared to matched parental cell lines [11,21-26]. The fact that this mutated EGFR variant has been found in tumors and not in normal tissues has made it an interesting target for antitumor therapies [27]. In this study, we aimed to estimate the proportion of EGFRvIII mutations in GBM patients in the Indian population. This study will contribute to the framing of targeted therapies in the future for these patients, which may improve their survival rate.

## Materials and methods

This study is an ambispective, single institutional observational study done on brain tissue biopsies of histopathologically diagnosed and confirmed cases of GBM based on the World Health Organization (WHO) 2007 Classification received in the Pathology Department of Dr. Sampurnanand Medical College, Jodhpur, from 2015 to 2020. The proposal for the study was reviewed thoroughly and approved by the Institutional Ethical Committee of Dr. Sampurnanand Medical College, Jodhpur (EC/MC/JU/2018/319) before the commencement of the study.

After applying the inclusion and exclusion criteria, as depicted in Table 1, paraffin-embedded tissue blocks of 35 cases of histopathologically confirmed GBM were retrieved. The epidemiological data of the selected cases were procured from the patient records maintained in the Pathology Department.

**Table 1:** Inclusion and exclusion criteria.

Inclusion criteria	Exclusion criteria
All brain biopsies with histopathological diagnosis of glioblastoma multiforme from 2015 to 2020	Extremely small biopsies (<5 mm)
	Glioblastoma multiforme cases with single tissue block
	Biopsies of the brain with histopathological diagnoses other than glioblastoma multiforme

Reverse transcription-polymerase chain reaction

Initially, excess paraffin was trimmed off the tissue block. Subsequently, 10-15 sections of 10 µ thickness were taken from each case, and the excess paraffin was trimmed using a sterile blade and placed in labeled Eppendorf tubes of 2 mL volume. RNA from these tissue sections was extracted using the ReliaPrepTM FFPE Total RNA Miniprep System (Promega, Madison, WI, USA) according to the manufacturer’s instructions. Initially, samples were deparaffinized using mineral oil, followed by sample lysis by adding lysis buffer and proteinase K. Then, the DNAase treatment mix was added to the sample. Further, isopropanol and buffer were incorporated for nucleic acid binding. A wash solution containing 95-100% ethanol was then used as a washing solution to obtain eluted RNA. It was stored at -30°C to -10°C in an elution tube. Extracted RNA concentration was analyzed on DS-11 from DeNovix (Wilmington, DE, USA). RNA samples with an adequate concentration were then processed for EGFRvIII detection using the TRUPCR EGFRvIII detection kit (3B Black Bio, Biotech India Pvt. Ltd.) according to the manufacturer’s protocol. An ABL1 primer-probe mix was taken as an internal control. The sample mixtures were then tested in the Microbiology Department (Dr. Sampurnanand Medical College) using reverse transcription-polymerase chain reaction (RT-PCR) (Bio-Rad, Hercules, CA, USA). The cycle threshold value (Ct value) was 33 for EGFRvIII mutation detection.

Statistical analysis was performed using the SPSS version 22.0 software package (IBM Corp., Armonk, NY, USA).

## Results

**Figure 1:**
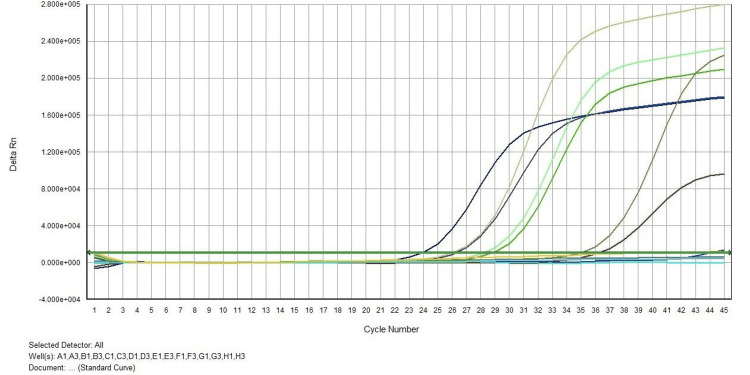
RT-PCR graph of tissue samples showing the delta run versus cycle number. RT-PCR: reverse transcription-polymerase chain reaction

Out of the total 35 cases, an EGFRvIII mutation was noted in 17.1% (6/35) cases (Figure 1). The mean age of presentation of EGFRvIII-positive cases was 54.3 years. Among positive cases, most were beyond 40 years of age (83.3%). Further, out of eight cases with an age less than 40 years, only one case was EGFRvIII positive (12.5%), while positivity was seen in five out of 27 cases beyond the 40-year age group (18.5%). As shown in Table 2, the majority of EGFRvIII-negative cases (37.9%) were 41 to 50 years old. However, the association between age and EGFRvIII positivity was not statistically significant (Fisher exact test, p-value = 1.000).

**Table 2:** Age distribution. P-value was 1.0000 for the association between age and EGFRvIII positivity. EGFRvIII: epidermal growth factor receptor variant III

Age (years)	EGFRvIII				Total	
	Positive		Negative			
	N	%	N	%	N	%
17–30	0	0.00	4	100.00	4	11.43
31–40	1	25.00	3	75.00	4	11.43
41–50	1	8.33	11	91.67	12	34.29
51–60	2	40.00	3	60.00	5	14.29
61–70	2	20.00	8	80.00	10	28.57
Total	6	17.14	29	82.86	35	100.00

In EGFRvIII-positive cases, males were affected twice as commonly as females, while the male-to-female ratio was 1.4:1 in EGFRvIII-negative cases. EGFRvIII positivity was more in males (19.05%) compared to females (14.29%); however, the difference was not statistically significant (p = 1.000) (Table 3).

**Table 3:** Gender distribution. EGFRvIII: epidermal growth factor receptor variant III

Gender	EGFRvIII				Total	
	Positive		Negative			
	N	%	N	%	N	%
Male	4	19.05	17	80.95	21	60.00
Female	2	14.29	12	85.71	14	40.00
Total	6	17.14	29	82.86	35	100.00

The association between gender and EGFRvIII-positive cases was not significant statistically (Fisher exact test, p-value = 1.000).

Most of the EGFRvIII-positive cases presented unilaterally (66.67%). The tumor was located in the right frontoparietal region in two cases, the left frontal region in one case, the left temporal region in one case, the right frontal and left parietal regions in one case, and the corpus callosal region in one case.

## Discussion

In this study, a total of six cases were found to be positive for the EGFRvIII mutation (17.1%), which is similar to the findings of another study that investigated 75 GBM cases and found eight cases positive for the EGFRvIII mutation (10.7%) [28]. All of these cases were aged over 40 years. They also used RT-PCR for the identification of this mutation. These results corroborate our findings. In our study, only one case was under 40 years of age. Das et al. also found EGFRvIII positivity in 12.2% of cases [29]. A mutation was found in one out of 13 cases (7.7%) in the 45-year age group, while five out of 37 cases were found positive in the >45-year age group. These findings are similar to our study findings. In both studies, no significant correlation was noted between age or gender with EGFRvIII mutation, which is in line with our study.

In an Indian study done by Jose et al. in 2020, mutated EGFRvIII was found in 57.5% of GBM cases, which is higher than our study finding [28]. The use of different molecular techniques may be the prime reason for such variation. They performed multiplex ligation-dependent probe amplification (MLPA) to identify this mutation. In a study by Perne et al. on a comparison between MLPA and RT-PCR, it was concluded that MLPA is a superior technique compared to RT-PCR in copy number quantification [30].

Rourke et al. performed a clinical trial on GBM patients with EGFRvIII-directed chimeric antigen receptor T cells on 369 patients with histologically confirmed GBM and tested for EGFRvIII using the next-generation sequencing (NGS) assay over the course of two years, of which 79 (21%) cases tested positive for EGFRvIII [31].

Sugawa et al. conducted a similar study using Southern blot analysis and discovered an EGFRvIII mutation in 17% of GBM cases [4]. Some researchers used more than one technique for estimating EGFRvIII positivity. Feldkamp et al. performed three techniques, namely, immunohistochemical testing, RT-PCR, and Western blot, and reported 25% EGFRvIII mutation positivity, which is in line with our results [32]. Thus, EGFRvIII mutation has been found in 10-50% of GBM cases worldwide in most studies.

The mean age of EGFRvIII-positive cases in our study was 54.3 years, while it was 56.1 ± 13.8 years in newly diagnosed GBM cases in the study by Shinojima et al. [33]. The mean age of GBM cases with EGFRvIII mutation was 47.13 years in the study by Jose et al. [28].

In our study, the male-to-female ratio was estimated to be 2:1 and 1.4:1 in EGFR-vIII positive cases aged ≤40 years and >40 years, respectively, while in the study of Jose et al., it was found to be 1:1 in ≤40 years and male predominance in >40 years of age, similar to the present study [28].

Studies done in the past on this molecular marker in GBM are listed in Table 4.

**Table 4:** Previous studies on epidermal growth factor receptor in glioblastoma multiforme patients.

Year	Authors	Study region	Technique used	Positivity
2020	Jose et al. [28]	India	Multiplex ligation-dependent probe amplification	57.5%
2017	Felsberg et al. [34]	Germany	Immunohistohemistry	50.5%
			Reverse transcription-polymerase chain reaction	56.8%
2017	O’Rourke et al. [31]	Philadelphia, United States	Next-generation sequencing	21%
2011	Jha et al. [35]	India	Reverse transcription-polymerase chain reaction	10.7%
2011	Montano et al. [36]	Italy	Reverse transcription-polymerase chain reaction	43.8%
2008	Yashimoto et al. [37]	California, United States	Reverse transcription-polymerase chain reaction)	27%
2008	Stutz et al. [38]	United States	Southern blot	17%
2007	Saikali et al. [39]	France	Immunohistochemistry and reverse transcription-polymerase chain reaction)	64%
2004	Aldape et al. [40]		Immunohistochemistry)	43.2%
			Reverse transcription-polymerase chain reaction	40.9%
2003	Shinojima et al. [33]	Japan	Immunohistochemistry)	25.3%
2000	Frederick et al. [41]	Minnesota	Sequence analysis	67%
1999	Feldkamp et al. [32]	United States	Reverse transcription-polymerase chain reaction, immunohistochemistry, and Western blot	25%
1995	Moscatello et al. [27]	Philadelphia	Western blot	56%
1990	Sugawa et al. [23]	Sweden	Southern blot	17%

The mean age of presentation in EGFR-negative cases in our study was 49.3 years, while it was 52 years in the study by Jose et al. [28]. In their study, Shinojima et al. reported the mean age of EGFRvIII-negative cases to be 51.0 ± 13.8 years, which is similar to our study. Most of the EGFRvIII-negative cases occurred beyond 40 years of age (75.9%), with the maximum cases between 41 and 50 years (91.7%) in our study.

The most common location in the brain involved by this tumor was the temporal region in the study by Jose et al., while the frontal lobe was found to be most commonly involved by EGFRvIII-mutated tumors in our study [28].

Study limitations

The principal limitations of this study were that patients were from a single center and the small sample size. Therefore, the findings cannot be readily generalized or considered conclusive unless confirmed by subsequent studies in our population. Moreover, limited clinical data restricted the subdivision of tumors into primary and secondary glioblastoma, which could have provided information regarding the correlation of EGFRvIII to subtypes.

## Conclusions

EGFRvIII detection is a major breakthrough in the improved management of GBM tumors through targeted gene therapies. Several clinical trials are underway globally to find a specific therapy against this mutation. However, research related to this mutation is way behind in India. Although extensive literature in Western countries is available on this marker, knowledge about this marker in the Indian population, particularly regarding brain tumors, is limited. Identification of this mutation may segregate patients who would be more responsive to newer therapeutic approaches. Previous studies also found that tyrosine kinase inhibitors only worked in cases of EGFRvIII mutations with a retained PTEN gene. The simultaneous presence of molecular markers in GBM cases would also need to be considered before initiating any gene therapy for its improved effectiveness.
